# One-year survival rate and factors associated with stage IV colorectal cancer at National General Hospital in Indonesia

**DOI:** 10.3389/fmed.2026.1781435

**Published:** 2026-03-05

**Authors:** Ardy Wildan, Eunike Vania Christabel, Ikhwan Rinaldi, Murdani Abdullah, Dadang Makmun

**Affiliations:** 1 Department of Internal Medicine, Cipto Mangunkusumo National General Hospital, Faculty of Medicine, Universitas Indonesia, Jakarta Pusat, Jakarta, Indonesia; 2 Division of Hematology and Medical Oncology, Department of Internal Medicine, Cipto Mangunkusumo National General Hospital, Faculty of Medicine, Universitas Indonesia, Jakarta Pusat, Jakarta, Indonesia; 3 Division of Gastroenterology, Pancreatobilliary, and Digestive Endoscopy, Department of Internal Medicine, Cipto Mangunkusumo National General Hospital, Faculty of Medicine, Universitas Indonesia, Jakarta Pusat, Jakarta, Indonesia

**Keywords:** colorectal cancer, stage IV colorectal cancer, one-year survival, associated factors, metastatic colorectal cancer

## Abstract

**Objective:**

To evaluate one-year survival and identify mortality-associated factors in stage IV colorectal cancer patients in Indonesia.

**Patients and methods:**

A retrospective cohort study was conducted using medical records of 214 patients aged ≥18 years who were diagnosed with stage IV CRC at Cipto Mangunkusumo National General Hospital between January 2018 and May 2020. Variables analyzed included demographic characteristics, body mass index (BMI), and chemotherapy status. Survival analysis was performed using the Kaplan–Meier method and Cox proportional hazards model.

**Results:**

Key findings included a one-year mortality rate of 53.3% and a median survival of 9.0 months (IQR 3.0–12.0). Most patients were under 60 years old (66.8%), and 47% were underweight (BMI < 18.5 kg/m^2^). Multivariate analysis revealed that a BMI < 18.5 kg/m^2^ (HR: 1.49) and lack of chemotherapy (HR: 4.47) significantly predicted increased mortality. Multivariate analysis revealed that a BMI < 18.5 kg/m^2^ (HR: 1.49) and lack of chemotherapy (HR: 4.47) were independently associated with higher one-year mortality. However, the observed survival difference related to chemotherapy may be influenced by patient selection and baseline clinical condition.

**Conclusion:**

This study demonstrates poor one-year survival among stage IV colorectal cancer patients in Indonesia. Underweight status and non-receipt of chemotherapy were independently associated with higher mortality; however, chemotherapy status likely reflects underlying patient fitness and disease severity rather than a direct causal effect. These findings highlight the challenges of late presentation, poor nutritional status, and limited access to systemic therapy in resource-limited settings.

## Introduction

Colorectal cancer (CRC) ranks as the third most prevalent cancer worldwide and is the second leading cause of cancer-related mortality. According to Global Cancer Statistics (GLOBOCAN) 2020, there were approximately 1.93 million new CRC cases reported, with one death occurring for every ten cancer cases (1). In 2016, data from the United States (US) showed that there were total incidences of 134,490 cases from 70,820 men and 63,670 women with total deaths due to CRC as many as 49,190 cases; 26,020 in men and 23,170 in women. In Asia, the highest incidence is found in the Eastern part of the continent with an incidence of 421,250 (2). This trend is believed to result from changes in lifestyle, including a diet rich in fat but low in fiber, along with smoking and lack of physical activity. Additionally, improvements in healthcare that contribute to increased life expectancy are expected to coincide with a rise in CRC cases (3).

The healthcare system of Indonesia faces a substantial challenge in the form of colorectal cancer (CRC). The mortality rate of colorectal cancer is significantly influenced by the disease stage at the time of diagnosis (4). Regrettably, the majority of patients in Indonesia only pursue therapy after their cancer has progressed to a more severe stage. Additionally, Indonesia currently lacks early detection and monitoring of colorectal cancer. The results of a study conducted in Semarang indicated that 16.7% of asymptomatic individuals had positive fecal occult blood tests (FOBT) (5). Of these patients, 32.4% were diagnosed with cancer or adenoma (pre-cancer). Moreover, the survival rate of CRC patients in Indonesia is currently the subject of limited evidence. According to the majority of studies, chemotherapy is the primary treatment option for patients with advanced colorectal cancer (6).

This study aimed to ascertain the one-year survival rate of metastatic colorectal cancer (mCRC) and to assess the factors influencing it in patients undergoing treatment at a national tertiary hospital, highlighting the importance of cancer survival analysis in the management of colorectal cancer (CRC). Age, body mass index (BMI), tumor laterality, metastatic location, and treatment are the aspects that should be prioritised as interconnected.

## Materials and methods

This was a retrospective cohort study using medical records of 214 patients treated at Cipto Mangunkusumo National General Hospital between January 2018 and May 2020. Inclusion criteria were patients aged ≥18 years with a confirmed diagnosis of stage IV colorectal cancer based on histopathology and evidence of metastasis from imaging or histopathology. The exclusion criterion was patients whose medical records could not be located.

We extracted data on demographic characteristics, such as age and sex, and clinical characteristics, such as BMI, history of colonoscopy, early symptoms, tumor location, serum carcino-embryonic antigen (CEA) level, tumor differentiation, metastatic site, and treatment given. The time when a patient first diagnosed with mCRC by radiological or histopathological finding was regarded as T0. Patients were followed until death or the end of one-year observation. Histopathological results were available for all patients, as histopathological confirmation was an inclusion criterion for this study. Data collected included tumor differentiation, T and N staging, and primary tumor location. While these variables helped characterize the study population and provided important context regarding disease severity, they were not significantly associated with one-year survival in our final multivariate analysis. Nevertheless, pathological features remain important prognostic factors and may guide risk stratification and treatment planning in clinical practice.

In bivariate analysis, we classified age into two groups, which were <60 years old and ≥60 years old according to elderly classification in Indonesia. We classified BMI to underweight (BMI < 18.5 kg/m^2^) and not underweight (BMI ≥ 18.5 kg/m^2^) to facilitate binary statistical analysis using Cox proportional hazards regression. This classification corresponds to the World Health Organization (WHO) cut-off for underweight, which has been associated with increased mortality risk in colorectal cancer patients according to previous studies (7).

Data were analyzed using IBM SPSS Statistics for Windows version 20 (SPSS Inc., Chicago, Illinois, USA). Numerical variables were presented as mean or median, while categorical variables were presented using proportion. One-year survival rate analysis was performed using Kaplan–Meier curve. Predictor variables with *p* < 0.25 in univariate analysis were entered to Cox proportional hazard regression model in order to obtain hazard ratio. Since this study was retrospective and only used existing medical record data, informed consent from patients was not required. This study was approved by the Ethics Committee of the Faculty of Medicine, Universitas Indonesia (approval number: ND237/UN2. F1/ETIK/PPM.00.02/2021, date: March 22nd, 2021).

Information on performance status (ECOG), comorbidities, molecular biomarkers (KRAS, NRAS, BRAF, MSI), socioeconomic status, and detailed metastatic burden was not consistently available in the medical records and therefore could not be included in the multivariate analysis. Chemotherapy was analyzed as a binary variable (yes/no), as treatment regimens, number of cycles, and treatment lines were heterogeneous and not uniformly documented. Survival outcomes in this study reflect overall mortality, as cause-specific mortality data were unavailable.

## Results

**Figure 1:**
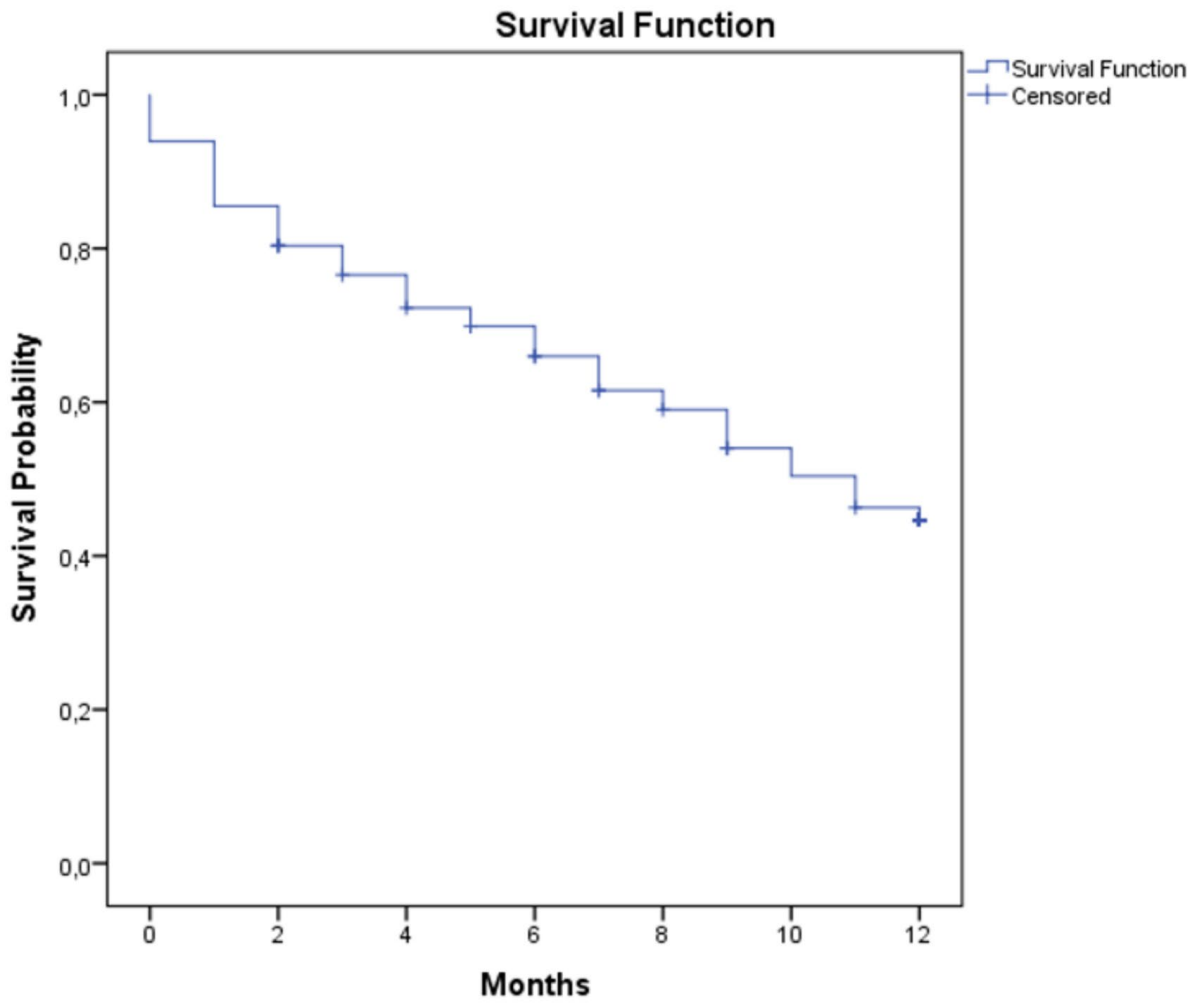
Kaplan–Meier survival curve.

A total of 228 patients met the inclusion criteria and were recruited as the study participants. 14 patients were subsequently excluded as the corresponding medical records could not be obtained, thus a total of 214 patients were included in the final analysis. The proportion of the male and female participants were similar, most of the participants (66.8%) were below 60 years of age. A total of 47% of the participants were underweight, determined as having BMI of <18.5 kg/m^2^ (see Table 1). Most study participants (57.9%) were diagnosed at stage IVA, with liver being the most common organ for metastasis (39.7%) (see Table 2). Mortality analysis demonstrated a one-year mortality rate of 53.3% with median survival duration of 9.0 (IQR 3.0–12.0) months (see Figure 1). All survival analyses were based on overall mortality during the one-year follow-up period; cause-specific mortality data were not available in the medical records.

**Table 1:** Baseline characteristics.

Characteristics	*N* = 214
Age (years old), median (IQR)	54.0 (40.7–62.0)
Sex, *n* Male (%)	109 (50.9)
BMI categories, *n* (%)	
Underweight (<18.5 kg/m^2^)	101 (47.2)
Normal (18.5–22.9 kg/m^2^)	68 (31.8)
Overweight (23.0–24.9 kg/m^2^)	25 (11.7)
Obese (>25.0 kg/m^2^)	20 (9.3)
Colonoscopy, *n* (%)	119 (59.5)
Early symptoms, *n* (%)	
Constipation	72 (33.6)
Hematochezia	68 (31.9)
Diarrhea	23 (10.7)
Abdominal pain	17 (7.9)
Combination	25 (11.8)
Primary tumor location, *n* (%)	
Rectum	135 (63.1)
Colon	79 (36.9)
Tumor side, *n* (%)	
Right sided tumor	32 (15.0)
Left sided tumor	177 (82.7)
Unspecified	5 (2.3)
CEA level, *n* (%), *N* = 121	
<5 mg/mL	19 (8.9)
≥5 mg/ml	102 (47.7)

BMI, body mass index; CEA, carcinoembryonic antigen; IQR, interquartile range.

**Table 2:** Tumor characteristics.

Characteristic	*n* (%)
Tumor differentiation, *n* (%)	
Well differentiated adenocarcinoma	81 (37.9)
Moderately differentiated adenocarcinoma	74 (34.6)
Poorly differentiated adenocarcinoma	30 (14.0)
Others	29 (13.6)
Tumor size, *n* (%)	
*T*2	10 (4.7)
*T*3	71 (33.2)
*T*4	122 (57)
*T*x	11 (5.1)
Lymph node involvement, *n* (%)	
*N*0	23 (10.7)
*N*1	123 (57.5)
*N*2	52 (24.3)
*N*x	16 (7.5)
Tumor staging, *n* (%)	
IVA	124 (57.9)
IVB	67 (31.3)
IVC	23 (10.7)
Metastatic organ involvement, *n* (%)	
Liver only	85 (39.7)
Lung only	20 (9.3)
Peritoneum only	12 (5.6)
Bone only	5 (2.3)
Ovarium only	6 (2.8)
Liver and lung	41 (19.2)
Lung and bone	8 (3.7)
Liver and peritoneum	6 (2.8)
Lung and peritoneum	4 (1.9)
Liver, lung, and bone	9 (4.2)
Liver, lung, and peritoneum	5 (2.3)
Liver, lung, and brain	5 (2.3)

A great proportion of the study participants (43%) received a combination therapeutic approach of surgery and chemotherapy. Of the 214 participants, a total of 90.2% of the participants underwent surgery, 61.7% received chemotherapy, and 10.3% received targeted therapy (see Table 3). Cox proportional hazard regression revealed that only BMI < 18.5 kg/m^2^ (HR: 1.49; 95%CI: 1.03, 2.17) and no chemotherapy (HR: 4.47; 95%CI: 3.03, 6.59) were linked to greater mortality rate (see Table 4).

**Table 3:** Treatment modalities.

Characteristic	*N* = 214
Treatment modalities, *n* (%)	
Surgery only	54 (25.2)
Surgery + chemotherapy	92 (43.0)
Surgery + radiation	7 (3.3)
Surgery + chemotherapy + targeted therapy	22 (10.3)
Surgery + chemotherapy + radiation	18 (8.4)
Supportive therapy	21 (9.8)
First line chemotherapy, *n* (%), *N* = 132	
Capecitabine only	21 (15.9)
CapeOX regimen	14 (10.6)
FOLFOX regimen	84 (63.6)
FOLFIRI regimen	9 (6.8)
FUFA regimen	3 (2.3)
FOLFOXIRI regimen	1 (0.8)
No chemotherapy	82 (41.0)
Second line chemotherapy, *n* (%), *N* = 38	
Capecitabine only	12 (31.6)
CapeOX regimen	2 (5.3)
FOLFIRI regimen	19 (50.0)
FOLFOX regimen	4 (10.5)
FOLFOXIRI regimen	1 (2.6)
Third line chemotherapy, *n* (%), *N* = 10	
Capecitabine only	3 (30.0)
CapeOX regimen	2 (20.0)
FOLFOX regimen	3 (30.0)
FOLFIRI regimen	1 (10.0)
FUFA regimen	1 (10.0)
Surgery, *n* (%), *N* = 193	
Elective	121 (62.7)
Emergency	72 (37.3)
Targeted therapy, *n* (%), *N* = 22	
Bevacizumab	12 (54.5)
Cetuximab	9 (40.9)
Cetuximab followed by bevacizumab	1 (4.5)
Metastatasis site specific treatment, *n* (%), *N* = 9	
Liver metastasectomy	6 (66.7)
RFA on liver nodule	3 (33.3)

**Table 4:** Factors associated with mortality in colorectal cancer patients.

Variable	Mortality *n* (%)		Crude HR (95%CI)	*p*	AHR (95%CI)	*p*
	Yes	No				
Age						
<60 years	80 (55.9)	63 (44.1)	1.24	0.30		
≥60 years	34 (47.9)	37 (52.1)	(0.83–1.85)			
BMI						
<18.5 kg/m^2^	65 (64.4)	36 (35.6)	1.76	**0.003**	1.49 (1.03–2.17)	**0.035**
≥18.5 kg/m^2^	49 (43.4)	64 (56.6)	(1.21–2.55)			
Tumor side						
Right sided tumor	19 (59.4)	13 (40.6)	1.23	0.42		
Left sided tumor	92 (52)	85 (48.0)	(0.75–2.01)			
Metastatic site						
Other than liver	74 (57.4)	55 (42.6)	1.26	0.23	1.25 (0.85–1.84)	0.26
Liver	40 (47.1)	45 (52.9)	(0.86–1.85)			
Chemotherapy						
No	68 (82.9)	14 (17.1)	4.70	**<0.001**	4.47 (3.03–6.59)	**<0.001**
Yes	46 (34.8)	86 (65.2)	(3.19–6.91)			
Targeted therapy						
No	110 (57.3)	82 (42.7)	4.45	**0.003**	2.27 (0.81–6.34)	0.12
Yes	4 (19.2)	18 (81.8)	(1.64–12.09)			

AHR, adjusted hazard ratio; BMI, body mass index; CI, confidence interval; HR, hazard ratio. Bold values indicate statistically significant results (*p* < 0.05).

## Discussion

The purpose of this study was to ascertain the one-year survival rate of patients with mCRC and the factors that are linked to it. We discovered that the median survival was 9 months, and the one-year survival rate was 46.7%. According to statistical research, a BMI below normal and the absence of chemotherapy were linked to a decreased chance of survival.

Our subjects’ median age was 54, with the majority being under 60. Other research examining the Indonesian population (8), but not in other Asian nations like China and Malaysia, also reported this feature, which showed that CRC was more common in younger people than in older people (9, 10). Although no research has looked into the source of this phenomena, the fact that early age has been linked to a worse prognosis should worry us (11). The percentage of male and female participants in our study was quite comparable by sex. However, some research found that male participants had a higher prevalence of colorectal cancer (CRC) than female ones, most likely as a result of estrogen’s protective action against CRC (9, 10, 12). Estrogen may exert its protective effect through the modulation of inflammation and inhibition of tumorigenesis, particularly via estrogen receptor beta (ERβ). However, ERβ expression declines with age and has been shown to be selectively lost in malignant colonic epithelial cells. This reduction in ERβ expression has been associated with colorectal cancer progression, which may help explain sex-based differences in CRC prevalence and outcomes.

Most of our participants developed tumors on the left side. According to some references, left-sided CRC is typically detected in younger people and is typically associated with liver metastases (13). Since none of our subjects had no symptoms, the majority were arriving late. In a study conducted in Semarang, Indonesia, 221 participants aged 45 and older who had no history or symptoms of CRC were screened. Remarkably, 3 and 2% of those participants, respectively, had pre-cancerous lesions and malignancy detected by the investigation (5).

In our study, the median survival was 9 months, and the one-year survival rate was 46.7%. The median survival for mCRC was 18 months (95%CI: 6.98, 29.02) in another Indonesian study (8). However, only 61.7% of the patients in our study underwent chemotherapy, whereas all individuals in that study did. In contrast to 36.4% of participants in our study, only 20.8% of subjects had metastases at two or more sites based on the number of metastatic organs. Although the reasons for this decreased survival were unknown, our median survival was lower than studies done in other nations that reported median survival >12 months (9, 14). Although chemotherapy is the standard treatment for metastatic colorectal cancer (mCRC), approximately 38% of patients in our study did not receive chemotherapy. This may be attributed to several factors, including poor performance status, late-stage presentation with extensive disease burden, comorbid conditions limiting therapy eligibility, financial constraints, or limited access to oncology services. Patient refusal or preference may also have contributed.

Chemotherapy was analyzed as a binary variable without accounting for differences in regimens, number of cycles, treatment intensity, or lines of therapy. Given the heterogeneity of chemotherapy protocols and non-standardized treatment pathways in routine clinical practice, this simplification may obscure important differences in treatment response and survival outcomes. Therefore, the magnitude of the observed association between chemotherapy and mortality should be interpreted with caution.

Most patients in our study presented at an advanced stage, which likely contributed to the poor survival outcomes. This may reflect delays in diagnosis due to limited awareness of early symptoms, restricted access to diagnostic services, and the absence of widespread colorectal cancer screening programs in Indonesia. A study in Semarang, Indonesia, found that among asymptomatic individuals undergoing fecal occult blood testing (FOBT), a substantial proportion had precancerous lesions or malignancy, emphasizing the potential impact of screening. Improving early detection and expanding access to screening may help improve survival outcomes for colorectal cancer patients in Indonesia (5).

The chemotherapy regimens in our study primarily included FOLFOX, Capecitabine, Capeox, and FOLFIRI, as recorded in the medical records. Treatment decisions were made based on the clinical condition of each patient, and regimens were not strictly standardized across the cohort. Targeted therapy, mainly bevacizumab, was given to a limited number of patients, reflecting access and resource constraints.

Only BMI < 18.5 kg/m^2^ (HR: 1.49; 95%CI: 1.03–2.17) and no chemotherapy (HR: 4.47; 95%CI: 3.03–6.59) were linked to greater mortality, according to our multivariate analysis. Large-scale investigations revealed that overall survival in advanced CRC patients with pre-diagnosis BMI < 18.5 kg/m^2^ was poorer than that of non-underweight subjects (HR: 2.0; 95%CI: 1.2, 3.2), despite conflicting findings describing the influence of underweight on CRC mortality (7, 15). Few research, including ours, have examined the impact of post-diagnosis underweight on mortality; nonetheless, a meta-analysis revealed that underweight also raised the risk of death for patients with CRC. Loss of muscle mass, which was correlated with the severity of the condition, was one of the potential causes (16). During the one-year observation period, 114 out of 214 patients (53.3%) died following the diagnosis of stage IV colorectal cancer. Although the overall mortality was recorded in this study, specific causes of death, were not documented in the medical records.

Non-receipt of chemotherapy was the strongest factor associated with increased one-year mortality in this cohort. While this finding is clinically plausible, it should be interpreted cautiously. In advanced colorectal cancer, chemotherapy eligibility is strongly influenced by baseline performance status, comorbidities, disease burden, nutritional status, and socioeconomic factors (22–24). As these variables were not available in our dataset, chemotherapy status in this study likely acts as a surrogate marker of patient fitness and disease severity rather than representing a direct causal treatment effect. Therefore, the observed association reflects real-world treatment selection rather than therapeutic efficacy.

Age, tumor side, and metastatic site, on the other hand, did not significantly correlate with survival. Other research revealed that survival varied with age, but it was still unclear which factor was associated with higher survival. While studies showing lower survival at older ages suggested that older patients had related degenerative processes, more comorbidities, higher procedure complications, and lower tolerance to chemotherapy, studies showing lower survival at younger ages contended that CRC at younger ages was more aggressive and less responsive to therapy (4, 6). Another study found that older age at diagnosis, rather than older chronological age, was associated with worse survival (17).

According to another study, the survival of right-sided CRC was lower than that of left-sided tumors (HR: 1.56; 95%CI: 1.43, 1.70) (18). This was most likely due to the flat morphology of right-sided CRC, which made it challenging to detect by colonoscopy. Right-sided CRC was frequently discovered at a later stage and lacked differentiation (13). However, due to the small number of participants with right-sided tumors, tumor side did not significantly increase the risk of death in our study.

At least 25–50% of individuals with CRC experienced liver metastases, making them the most frequent distant metastatic location (19). Lung-only metastasis was a favorable predictor for survival in a research that compared liver-only and lung-only metastases (HR: 0.69; 95%CI: 0.62, 0.76) (20). Fortunately, overall survival may be considerably increased in patients with CRC if liver metastases are removed (21). Because only 4.2% of the participants in our study received therapy for liver metastases, liver metastasis did not appear to be a substantial cause of death.

This study has several limitations. It was conducted using a retrospective cohort design, and the analysis relied on medical records, which may have contained incomplete or missing data such as CEA level, ECOG performance status, comorbidities, molecular biomarkers, surgical details, and diagnostic delays. These variables were not excluded intentionally but were unavailable in the records. We also did not examine the subjects directly, which may have led to differences in clinical data interpretation and introduced bias. As with any retrospective study, our analysis is subject to selection bias because only patients with complete and available medical records were included. Furthermore, unmeasured confounders, such as socioeconomic factors, detailed comorbidities, molecular markers, and performance status, may have influenced survival outcomes but could not be accounted for. The relatively small sample size and single-center design may have limited the statistical power to detect associations between some variables and survival, and may limit the generalizability of our findings. This could reduce the strength of the conclusions drawn, and caution is warranted when interpreting the results. Future prospective studies with larger samples and more comprehensive data collection are needed to validate these findings.

Despite these limitations, this study has strengths as it is the first cohort study in Indonesia to assess one-year survival in stage IV colorectal cancer. The study provides valuable data on the characteristics of stage IV colorectal cancer patients at our national referral hospital (RSCM), the treatments received, and their impact on patient survival. We also evaluated various factors influencing one-year survival. These findings are expected to help healthcare providers identify areas that need improvement to enhance survival outcomes for stage IV colorectal cancer patients at our center. In addition, this study can provide input for policymakers in developing strategies to improve colorectal cancer survival rates in Indonesia.

Several established prognostic factors in metastatic colorectal cancer, including ECOG performance status, comorbidities, molecular biomarkers (KRAS, NRAS, BRAF, MSI), and extent of metastatic disease, were not included in the multivariate model due to data unavailability. This substantially limits the interpretability of the adjusted hazard ratios, and residual confounding is likely. Consequently, the multivariate model should not be interpreted as a comprehensive prognostic assessment, but rather as an exploratory analysis based on variables available in routine clinical records.

Although this study aimed to evaluate a broad range of demographic, pathological, and treatment-related factors, only BMI and chemotherapy status remained independently associated with one-year mortality in the adjusted analysis. Other variables, while clinically relevant, did not retain statistical significance, likely due to residual confounding, sample size limitations, and incomplete data.

## Conclusion

In this cohort of stage IV colorectal cancer patients from a national referral hospital in Indonesia, one-year survival remained poor, with a median survival of 9 months. Underweight status and non-receipt of chemotherapy were independently associated with higher overall mortality; however, chemotherapy status likely reflects baseline patient fitness and disease severity rather than a direct causal effect. These findings underscore the challenges of late-stage presentation, poor nutritional status, and limited access to systemic therapy in resource-limited settings. Future prospective studies incorporating performance status, comorbidities, molecular profiling, and detailed treatment data are needed to better define prognostic factors and guide clinical decision-making.

## Data Availability

The original contributions presented in the study are included in the article/supplementary material, further inquiries can be directed to the corresponding author.
